# Mechanisms Controlling Anaemia in *Trypanosoma congolense* Infected Mice

**DOI:** 10.1371/journal.pone.0005170

**Published:** 2009-04-13

**Authors:** Harry A. Noyes, Mohammad H. Alimohammadian, Morris Agaba, Andy Brass, Helmut Fuchs, Valerie Gailus-Durner, Helen Hulme, Fuad Iraqi, Stephen Kemp, Birgit Rathkolb, Eckard Wolf, Martin Hrabé de Angelis, Delnaz Roshandel, Jan Naessens

**Affiliations:** 1 School of Biological Sciences, University of Liverpool, Liverpool, United Kingdom; 2 Faculty of Life Sciences, University of Manchester, Manchester, United Kingdom; 3 School of Computer Science, University of Manchester, Manchester, United Kingdom; 4 Immunology Department, Pasteur Institute of Iran, Tehran, Iran; 5 International Livestock Research Institute, Nairobi, Kenya; 6 GMC at the Helmholtz Zentrum München, Munich/Neuherberg, Germany; 7 Chair for Molecular Animal Breeding and Biotechnology, Gene Center, LMU Munich, Munich, Germany; 8 Chair for Experimental Genetics, Center of Life and Food Science Weihenstephan, Technische Universität München, Freising, Germany; INSERM U567, Institut Cochin, France

## Abstract

**Background:**

*Trypanosoma congolense* are extracellular protozoan parasites of the blood stream of artiodactyls and are one of the main constraints on cattle production in Africa. In cattle, anaemia is the key feature of disease and persists after parasitaemia has declined to low or undetectable levels, but treatment to clear the parasites usually resolves the anaemia.

**Methodology/Principal Findings:**

The progress of anaemia after *Trypanosoma congolense* infection was followed in three mouse strains. Anaemia developed rapidly in all three strains until the peak of the first wave of parasitaemia. This was followed by a second phase, characterized by slower progress to severe anaemia in C57BL/6, by slow recovery in surviving A/J and a rapid recovery in BALB/c. There was no association between parasitaemia and severity of anaemia. Furthermore, functional T lymphocytes are not required for the induction of anaemia, since suppression of T cell activity with Cyclosporin A had neither an effect on the course of infection nor on anaemia. Expression of genes involved in erythropoiesis and iron metabolism was followed in spleen, liver and kidney tissues in the three strains of mice using microarrays. There was no evidence for a response to erythropoietin, consistent with anaemia of chronic disease, which is erythropoietin insensitive. However, the expression of transcription factors and genes involved in erythropoiesis and haemolysis did correlate with the expression of the inflammatory cytokines *Il6* and *Ifng*.

**Conclusions/Significance:**

The innate immune response appears to be the major contributor to the inflammation associated with anaemia since suppression of T cells with CsA had no observable effect. Several transcription factors regulating haematopoiesis, *Tal1*, *Gata1*, *Zfpm1* and *Klf1* were expressed at consistently lower levels in C57BL/6 mice suggesting that these mice have a lower haematopoietic capacity and therefore less ability to recover from haemolysis induced anaemia after infection.

## Introduction

African trypanosomes are protozoan parasites that cause severe diseases in humans and livestock with fatal consequences unless treated. Whilst trypanosomiasis due to *Trypanosoma brucei spp* causes significant morbidity and mortality in humans, trypanosomiasis caused by *T. congolense* and *T. vivax* is one of the most significant constraints on cattle production in Africa and the cause of major economic losses with serious effects on human health and welfare [1].

African trypanosomes are extracellular parasites that survive in the blood stream. In cattle, anaemia is the key feature of disease and persists after the first wave of parasitaemia when parasite numbers have declined to low or undetectable levels. Anaemia rather than parasitaemia is best correlated with productivity and is used as the primary indicator of when to treat the infection [2]. Treatment to clear the parasites usually resolves the anaemia. Trypanotolerance, or the capacity of some ancient West African cattle breeds such as the N'dama to remain productive despite being infected, is correlated with a genetic capacity to limit anaemia [3], [4], [5]. In breeds that have been introduced to the continent more recently, such as the Boran, erythrocyte counts continue to decrease after parasitaemia has been controlled and, unless treated, the animals die with very low Packed Cell Volume (PCV).

Because of the importance of the anaemia in trypanosomiasis many studies have been carried out to describe its nature and discover its causes. The major mode of red blood cell elimination appears to be extravascular destruction due to a massive erythrophagocytosis in spleen and liver [6]. The observation of hyperactivated macrophages and erythrophagocytosis in tissues of infected cattle [7] suggests that they may be a major cause of anaemia and haemophagocytic syndrome [4]. However, evidence has been provided for the contribution of other mechanisms in different host-parasite combinations, such as haemolysins (reviewed in [6]), differences in type and amounts of sialic acids [8], [9], binding of autologous or polyreactive antibodies or complement C3 to erythrocyte surfaces [10]–[12] or the passive absorption of trypanosome molecules in the erythrocyte membrane [13]. Yet, immunological competence is not essential for the development of anaemia. Irradiated rats still became anaemic after *T. brucei* infection [5], [6] and *in vivo* T-cell depletion did not affect anaemia in cattle [14], [15].

Anaemia is also a feature in some murine trypanosomiasis models [16]–[20]. A comparison of anaemia and parasitaemia between A/J and more resistant C57BL/6 mice revealed that anaemia development was more severe in the C57BL/6 strain, despite the fact that this strain acquired lower parasitaemias and survived longer after infection than A/J mice [17]. A comparison of different host-parasite combinations revealed no correlation between pathology and survival [19]. Such data suggest that anaemia is a consequence of host responses to the infection, and not directly induced by the parasite products. Studies with *T. brucei* infected C3H/He mice suggested an involvement of nitric oxide (NO) [20]. In some murine models, but not others, anaemia was mediated by TNF [18], which seems to achieve its function via binding with TNFR2 [19]. And an extensive evaluation of anaemia related genes responding to infection of C57BL/6 mice with *T. brucei* was interpreted as evidence for increased iron storage and reduced erythropoiesis as a consequence of restricted iron availability [16].

In order to assess the parameters that influence anaemia in murine *T. congolense* infections we compared survival, parasitaemia and development of anaemia and pathology in three mouse strains A/J, BALB/c and C57BL/6 that differ in their susceptibility to trypanosomiasis with gene expression data from Affymetrix microarrays.

## Materials and Methods

### Animals

C57BL/6JOlaHSD, BALB/cOlaHsd, A/JOlaHsd mice (hereafter C57BL/6, BALB/c and A/J) were purchased from Harlan UK. Mice were kept in the small animal unit of the ILRI institute and treated in accordance with the Institute's Animal Care and Use Committee (IACUC) policies.

12 weeks old A/J, BALB/c and C57BL/6 mice were infected with 10^4^ *T. congolense* IL1180 parasites [18]. Parasites per ml of tail blood were enumerated using a haemocytometer. Mice were killed by cervical dislocation or CO_2_ euthanasia at appropriate time points post infection and spleen, liver and kidney were collected into liquid nitrogen.

The role of T cells in the response to infection was determined by treating six C57BL/6 mice with Cyclosporin A (CsA) and following the course of infection with *T. congolense* clone IL1180. CsA was a gift from Sandoz Ltd, Basel, Switzerland, and was solubilised in pure ethyl alcohol at 10 mg/ml and diluted in sterile saline (0.9% NaCl). A volume of 200 µl containing 400 µg CsA/mouse (about 20 mg/kg) was injected ip every other day for 10 days. Four control mice were injected with the same diluent without CsA.

### Blood parameters

Blood samples were tested for erythrocyte counts and relative haemoglobin concentration. Erythrocyte numbers were enumerated by haemocytometer under phase-contrast microscopy. The haemoglobin concentrations were measured spectrophotometrically at 540 nm [21]. Samples of 2 µl of blood were collected from the tail and diluted in 150 µl of distilled water in a plate with 96 round bottom wells (Costar 3799, Corning Incorporated, Corning NY, USA). After 30 minutes at room temperature, the plate was centrifuged at 600×g for 10 minutes, after which 100 µl of supernatant was transferred to a new plate and the optical density measured at 540 nm in an ELISA plate reader (Multiscan MCC/340, Titertek Instruments, Huntsville, AL, USA). Measurements were carried out in triplicate.

Additionally, blood was collected post mortem by opening the thoracic cavity, removing the sternum, cutting the vena cava caudalis and the aorta cranial to the diaphragm and collecting the leaking blood from the thoracic cavity using a pipette. Blood was left for two hours at room temperature to clot, then stirred and centrifuged at 4600×g for 10 minutes. The serum was collected, frozen and sent to the German Mouse Clinic (GMC). Iron metabolism related serum parameters (ferritin, transferrin) were determined from serum samples by the clinical chemical laboratory of the GMC using an AU 400 autoanalyzer (Olympus, Germany) and Olympus kits developed for the analysis of human samples that had been adapted for analysis of small volume mouse samples.

Analysis of variance (ANOVA) was performed on anaemia parameters (RBC counts and relative haemoglobin titres) and tissue weights using Genstat 11 software, with fixed effects for mouse genotype, days post infection (dpi) and genotype x dpi. For organ weight analysis, data were log transformed before analysis for dpi, strain, sex and interactions between them, sex x dpi, strain x dpi and strain x sex.

### Extraction of total RNA and Microarrays

*Ex vivo* tissue samples were ground under liquid nitrogen prior to RNA extraction. RNA was extracted from tissue with Trizol reagent (Invitrogen). RNA quantity and quality was determined with an Agilent 2100 bioanalyzer. Pools of RNA from five mice were prepared for hybridisation to Affymetrix 430_2 arrays. For each condition five independent pools of five RNA samples were hybridised to the arrays. The Affymetrix 430_2 arrays contain 45,000 probe sets for over 39,000 transcripts and variants from over 34,000 mouse genes. Labeling and hybridisation was done according to the manufacturers instructions.

Technical quality control was performed with dChip (V2005) (www.dchip.org) [22] using the default settings. Background correction, quantile normalization, and gene expression analysis were performed using RMA in BioConductor [23]. Statistical tests were conducted in SPSS (V16).

All microarray data has been deposited at ArrayExpress under the accession numbers E-MEXP-1190. The expression data and plots like those presented here are also available for all genes on the microarrays from the authors' website “Expression viewer” at http://www.genomics.liv.ac.uk/tryps/resources.html.

## Results

### Survival

**Figure 1:**
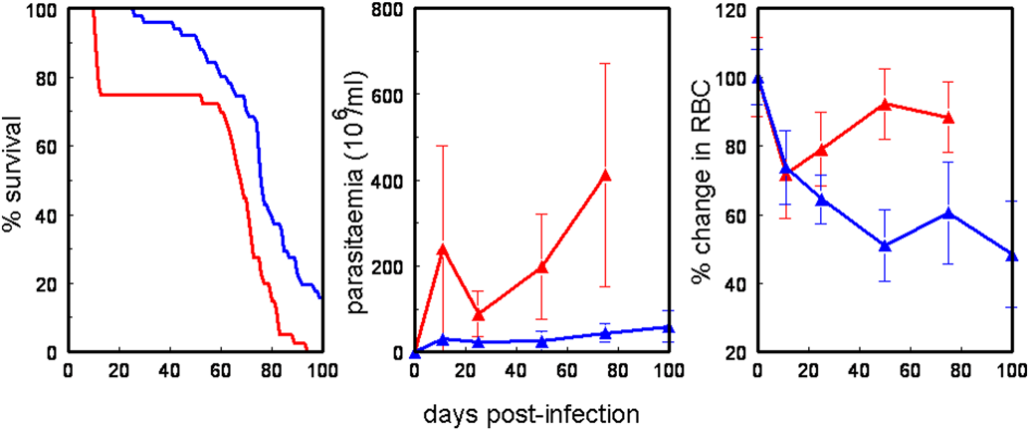
Comparison of survival (A), parasitaemia (B) and % change in red blood cell numbers (C) between susceptible A/J mice (red) and resistant C57BL/6 mice (green) after infection with a *T. congolense* IL1180, shown as mean±SD. The plots show that A/J mice have a shorter survival time (mean 57 days) than C57BL/6 (mean 71 days), higher parasitaemia but less severe anaemia.

As described previously [17], C57BL/6 mice survived a mean of 25 days longer than A/J mice after *T. congolense* infection (p = 0.04 Log Rank Survival Test; Fig. 1A). About 25% of A/J mice died around day 10–11 post-infection. This corresponds to the time when the first parasitaemic wave reached a peak. A second wave of mice died after day 50, again when parasitaemia increased to very high levels. BALB/c mice were not included in this experiment but they have a survival time that is intermediate between A/J and C57BL/6 mice [24]–[27]. *T. congolense* IL1180 is a relatively low virulence strain with long survival times after infection. *T. congolense* Tc13, which has been used in many other studies, kills BALB/c mice in about 12 days [28], therefore the responses to the two parasite strains may not be comparable.

### Parasitaemia

The first wave of parasitaemia peaked around day 9 (Fig. 1B) and parasitaemia was higher in A/J than in C57BL/6 mice.

### Anaemia

The change in red blood cell counts (RBC) was expressed as a percentage change of the pre-infection number, as uninfected A/J mice had a higher erythrocyte density. The number of RBC in the blood decreased after infection in both mouse strains (Fig. 1C). However, A/J mice recovered quickly after the first parasitaemic wave, while RBC numbers in C57BL/6 mice kept falling with time. C57BL/6 mice started dying when RBC density dropped below 60% of its normal value, suggesting that death in C57BL/6 mice was correlated with severe anaemia.

### Relative haemoglobin concentration

**Figure 2:**
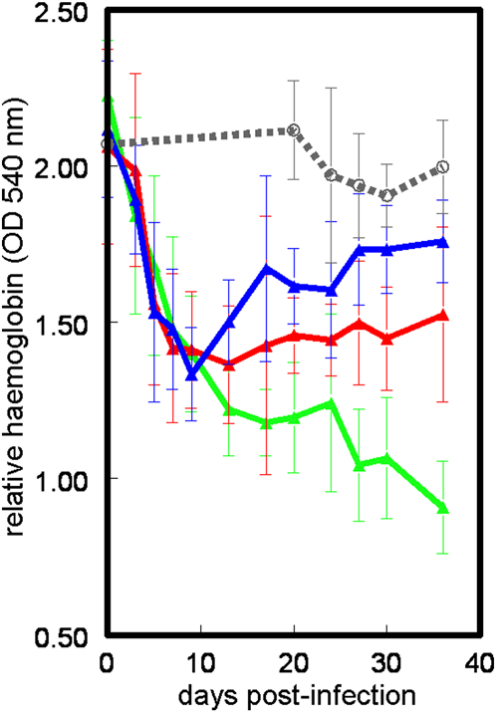
Haemoglobin titres in C57BL/6 (green), A/J (red) and BALB/c mice (blue) after infection with *T. congolense*, and uninfected C57BL/6 mice (grey, broken line), shown as mean±SD. Each point is an average of ten mice. Haemoglobin declines rapidly in all mouse strains until the first peak of parasitaemia after which it recovers to almost baseline levels in BALB/c mice, partially recovers in A/J mice but continues to decline in C57BL/6 mice.

Three additional *T. congolense* infection experiments were carried out in order to compare anaemia development between susceptible A/J, tolerant C57BL/6 and intermediately susceptible BALB/c mice. Mean haemoglobin titres were measured in ten mice of each strain up to day 30 post infection (pi). After infection, a decrease in haemoglobin titres was almost immediately noticeable in all three mouse strains (Fig. 2). This initial anaemia reached a nadir by day 10 pi in A/J mice and haemoglobin titres partially recovered thereafter. C57BL/6 mice never recovered and developed severe anaemia. The haemoglobin titre in BALB/c mice recovered even faster than the titre in A/J mice. Comparison of the mean haemoglobin titres in the three mouse strains on day 17 pi in three different infection experiments (Table 1) confirmed that C57BL/6 mice are the most susceptible to anaemia development, while BALB/c mice are the most resistant. In a previous evaluation of anaemia in A/J and C57BL/6 over a shorter time period (18 days) we also found that A/J had higher haemoglobin titres than C57BL/6 [29]. However, in that case A/J had higher titres from the start and the differences remained constant over the 18 days of the experiment. The difference in timing and size of the relative haemoglobin levels between strains between experiments may be due to the high variability in haemoglobin levels (note the large error bars in figure 2) but in both cases C57BL/6 developed a more severe anaemia than A/J.

**Table 1:** Mean relative haemoglobin titres (OD at 540 nm) and standard deviation in blood from susceptible A/J, intermediately susceptible BALB/c and more resistant C57BL/6 mice 17 days post-infection in three *T. congolense* infection experiments (n = 10 per experiment).

Mouse strain	A/J	BALB/c	C57BL/6
**Infection 1**	0.23±0.13	0.31±0.04	0.21±0.06
**Infection 2**	0.25±0.01	0.33±0.06	0.22±0.05
**Infection 3**	0.39±0.13	0.45±0.08	0.32±0.03
**Uninfected**	0.53±0.06	0.53±0.05	0.55±0.06

In each experiment, haemoglobin titres remained highest in BALB/c and fell to the lowest level in C57BL/6.

### Role of T lymphocytes in anaemia development

**Figure 3:**
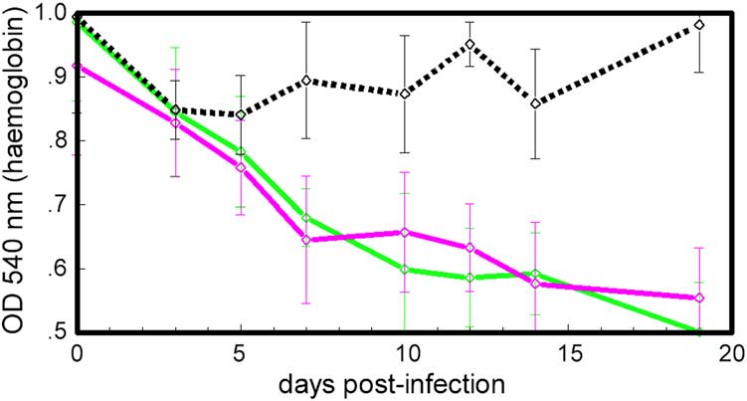
Mean haemoglobin titres in four C57BL/6 mice (green) and six CsA-treated C57BL/6 mice (magenta) after infection with *T. congolense*, and four uninfected C57BL/6 mice (black, broken line), shown as mean±SD. CsA induces defective T cells. Since there was no difference in anaemia after CsA treatment it is unlikely that T cells play a major role in the development of anaemia in C57BL/6 mice.

To find out whether T lymphocytes play a role in the development of anaemia, haemoglobin levels were compared between control and CsA-treated C57BL/6 mice. CsA is an immunosuppressive agent that induces defective T cells. No significant difference was observed in anaemia development between the two groups of mice (Fig. 3).

### Hepato-splenomegaly

Haematopoiesis normally occurs in the bone marrow. However, in any situation in which the number of blood cells significantly decreases and bone marrow cannot compensate for this loss alone, haematopoiesis may occur in extramedullary (outside bone marrow) sites including both liver and spleen [30], [31].

**Figure 4:**
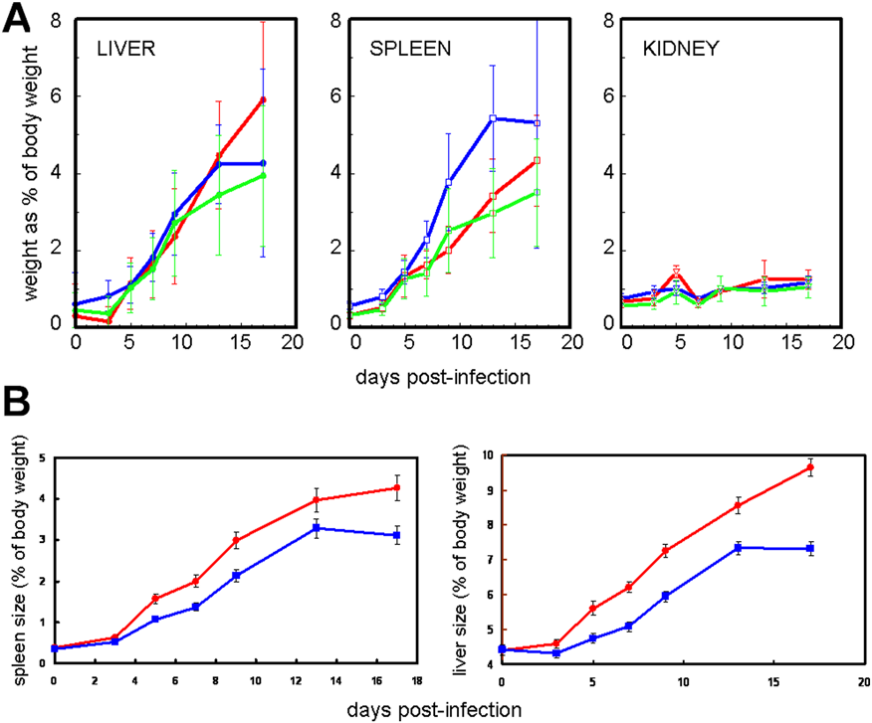
(A) Mean weights of internal organs relative to body weight during *T. congolense* infection in A/J mice (red), BALB/c mice (blue) and C57BL/6 (green) mice, shown as mean±SD. The mean relative weights of liver, spleen and kidney increased 1.9, 10.3 and 1.7 fold over the course of the infection (p<0.001). There were statistically significant (ANOVA p<0.05) differences in weight between strains at most time points but the largest and perhaps biologically most significant difference was in the spleen where the relative weight in BALB/c mice increased 12 fold and in A/J and C57BL/6 mice it increased about 9.4 fold. (B) The increase in mean spleen and liver weights (±StErr) is higher in female (red, circles) than male (blue, squares) mice (p<0.001).

To see whether there was a correlation between anaemia and hepato-splenomegaly, the weights of liver, spleen and kidney were monitored in all three mouse strains during the first 17 days of infection. Weights of liver, spleen and kidney increased 1.9, 10.3 and 1.7 fold respectively over this period, and the change in weight, both absolute and relative to body weight, was highly significant in all cases (ANOVA p<0.001) (Figure 4a). The increase in liver and spleen weights, but not kidney, was significantly (p<0.001) higher in females than in males (Figure 4b). There was a significant difference between strains in spleen weight (ANOVA p< = 0.005) pre-infection and at each sampling day post infection except day 5. BALB/c had the highest weight at all days; this may be associated with particularly high haematopoietic potential in this organ in this strain. There were also significant differences in liver, but not in kidney weight between strains (p<0.05) at most time points. However, the differences in weight were not large and may represent differences in timing of responses as much as fundamental differences in response. Total bodyweight increased slightly over the course of the infection but by less than the total increase in organ weight. This may reflect a loss of muscle mass and be a consequence of the cachexia that is a well-known consequence of the disease.

### Anaemia related metabolites

**Figure 5:**
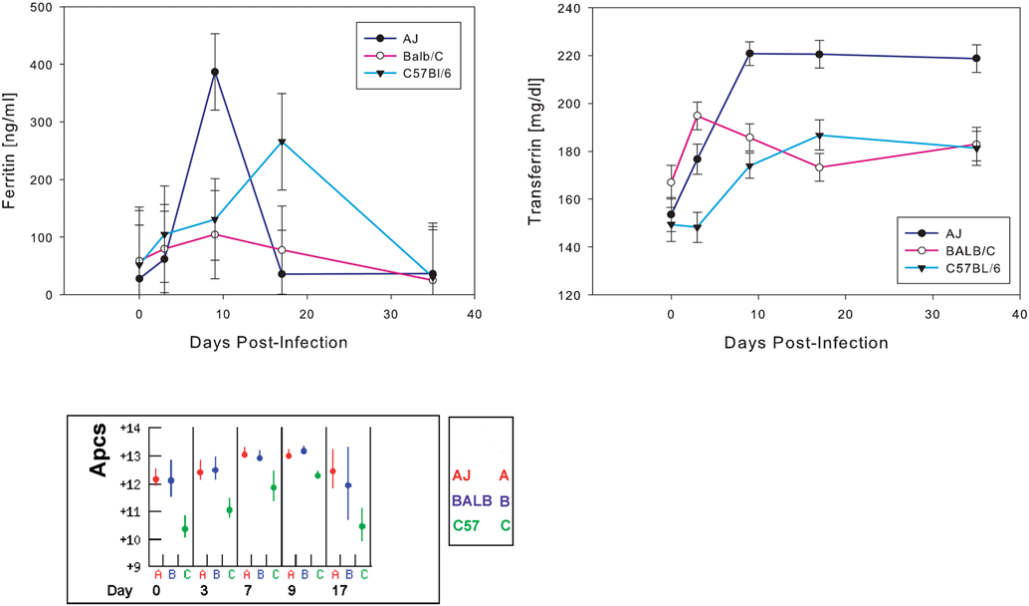
Acute phase proteins and ferritin. Titres of ferritin (A) and transferrin (B) in plasma from *T. congolense*-infected A/J, BALB/c and C57BL/6 mice, shown as mean±SD. (C) Expression of serum amyloid P (*Apcs*), the major murine acute phase protein, in the liver post infection.

The measurement of serum iron was precluded by high levels of haemolysis after infection. Ferritin levels did not differ significantly between strains or over time, due to a very high variance. However the mean values increased from day 0 to day 9 in all three strains, as it can be expected in haemolytic anemia. By day 17 ferritin concentration was declining in A/J and BALB/c mice but it increased further in C57BL/6 mice; all three strains showed normal values at day 35. Transferrin levels increased in all strains after infection (Fig 5a) and stayed relatively constant from day 3 (BALB/c) or day 9 (A/J and C57BL/6). The largest increase was seen in A/J mice.

### Genes regulating haematopoiesis

A large microarray gene expression data set was reviewed in order to identify the role of haematopoietic genes in the development of the differences in anaemia between the strains.

**Figure 6:**
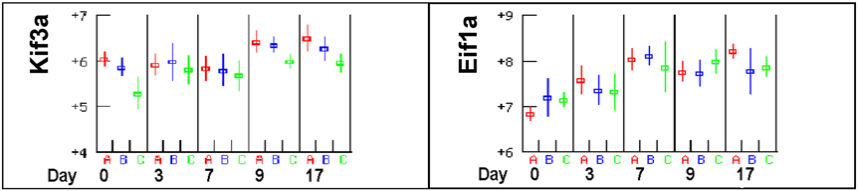
EPO responsive genes. *Kif3a* and *Eif1a* are EPO responsive genes but respond to inflammatory signals as well. Consequently their positive response to infection may not be related to induction of erythropoiesis.

The primary regulator of normal erythropoiesis is erythropoietin (EPO) expressed in the kidney. The erythropoietin gene was not included in the Affymetrix array. However genes that respond to erythropoietin were included; *Ptp4a1* (*Prl1*) and *Tnfrs11a* (*Rank*) have both been shown to respond to EPO and may further propagate EPO signals [32]. Neither of these genes appeared to respond to infection or to be differentially expressed, although *Ptp4a1* was highly expressed throughout the infection (not shown). Other *Epo* responsive genes did appear to respond to infection: *Eif1a* (Eukaryotic translation initiation factor) and *Kif3a* (kinesin family member 3A) were up regulated in all three mouse strains at day 7 and 9 respectively (Fig 6). However, since these genes participate in multiple signal transduction pathways, their up-regulation may be related to inflammation rather than erythropoiesis [32].

Erythropoietin primarily acts through the erythropoietin receptor (*Epor*) that is exclusively expressed on cells from the erythroid lineage. So the level of expression of *Epor* may be related to the number of erythroid cells in the tissues. In uninfected mice, *Epor* transcription was highest in A/J mice. After infection C57BL/6 mice tended to have lower levels of expression than either A/J or BALB/c mice although this was only significant if the expression levels were compared over the whole time course (p = 0.00003 with respect to BALB/c mice and p = 0.043 with respect to A/J mice). This was consistent with lower erythropoiesis and haemoglobin titre in C57BL/6 mice. However, given the small difference in expression and the lack of evidence for changes in *Epo* response genes this may not be an important mechanism driving anaemia after *T. congolense* infection.

**Figure 7:**
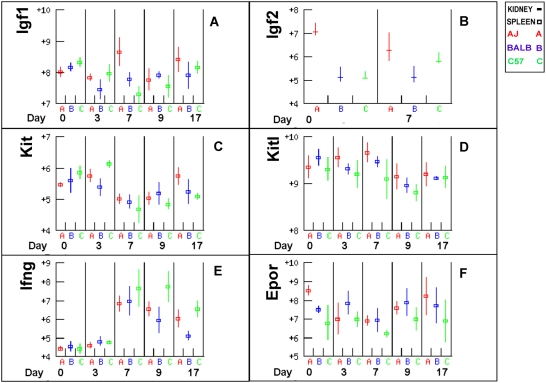
Genes that mediate haematopoiesis. *Igf1* appears to be an important regulator of erythropoiesis in some anaemic patients. *Epor*, *Kit* and *Kitl* are regulators of erythropoiesis. *Ifng* down-regulates *Epor* and *Kitl* and the expression of the three genes was consistent with this relationship.

Interferon gamma (*Ifng*) down regulates *Kit* ligand (*Kitl*) and *Epor* and may be an important contributor to anaemia of infection [33]. *Kit* (CD117) is a receptor for *Kitl* (SCF or Stem Cell Factor), which acts synergistically with *Epo* in the promotion of erythropoeisis [33]. *Ifng* expression increased approximately 8-fold after infection in all mouse strains and then declined fastest in BALB/c and remained highest in C57BL/6 consistent with the observed haemoglobin titres (Fig 7). Consistent with the inhibitory activity of IFN-γ, the expression of *Kit* and *Epor* all declined somewhat at day 7 pi and C57BL/6 had the lowest levels of expression after day three (Fig 7). Higher levels of *Kitl* may be indicative of higher levels of erythropoiesis. In the spleen *Kitl* expression was highest in A/J and lowest in C57BL/6 from day 3 to 9 (Fig 7).

Insulin like growth factor (*Igf1*) appears to be more important than *Epo* for regulation of erythropoiesis in some anaemic patients [34], [35], [36]. Expression of *Igf1* declined in C57BL/6 mice till day 7 pi, while it increased in A/J mice and was more than twice as high as in C57BL/6 on day 7 (Fig 7). However, differences at other days were small and no correlations with anaemia could be made. *Igf2* is the predominant regulator of erythropoietin-independent erythroid colony formation by neonatal progenitor cells and has antiapoptotic effects. Its expression was approximately 2–4 fold higher in A/J than C57BL/6 or BALB/c in the kidney, consistent with better recovery of A/J but not BALB/c from the initial drop in haematocrit.

Latexin has been found to be a negative regulator of the size of the haematopoietic stem cell population in mice [37]. Latexin expression increased in the liver of all strains by 40–100% at day 7–9 and decreased but remained above baseline levels at day 17 (not shown). The expression levels in the spleen were higher than in the liver but did not vary.

The CD34 membrane antigen is specifically expressed by activated haematopoietic stem cells [38]. Therefore, higher levels of CD34 signify the presence of higher numbers of haematopoietic stem cells, and could be an activity index for haematopoiesis. CD34 was approximately 30% more highly expressed on day 9 post-infection in both liver and spleen of A/J in comparison with C57BL/6 mice (not shown), suggesting higher levels of haematopoiesis in A/J.

**Figure 8:**
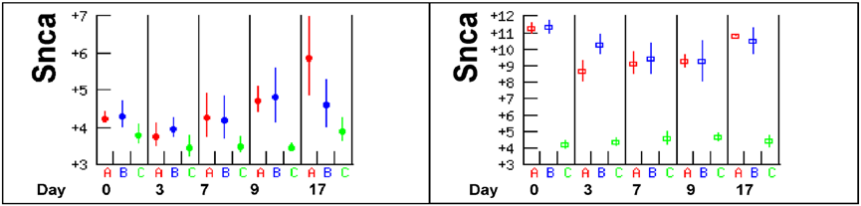
Expression of *Snca* in (A) liver and (B) spleen. *Snca* is strongly associated with reticulocytes and was the gene with largest expression difference that correlated with anaemia response. The strong expression of *Snca* in the spleen of A/J and BALB/c is suggestive of extra medullary haematopoiesis in this organ in these strains.

The function of synuclein-alpha (*Snca*) in erythropoiesis is not known, however an analysis of its expression in 71 tissues and cell types showed that it is expressed at maximum levels in early erythroid CD71 cells (reticulocytes) and in a separate analysis of human reticulocytes *Snca* was found in the top twenty most highly expressed genes [39], [40]. *Snca* was one of the genes with the greatest difference in mRNA abundance between C57BL/6 mice and the other two mouse strains, A/J and BALB/c, which had 60–250 fold higher expression of *Snca* than C57BL/6 mice in the spleen at all time points (Fig 8b). In the liver *Snca* expression levels were similar in all three strains until day 9 when the expression was about two fold higher in A/J and BALB/c mice than in C57BL/6 mice (Fig 8a). The transient increase in the liver could have been caused by circulating reticulocytes in anaemic animals, however the gross differences in expression in the spleen, even prior to infection, are suggestive of substantial differences in extramedullary haematopoiesis in the spleen.

### Transcription factors regulating erythropoiesis

**Figure 9:**
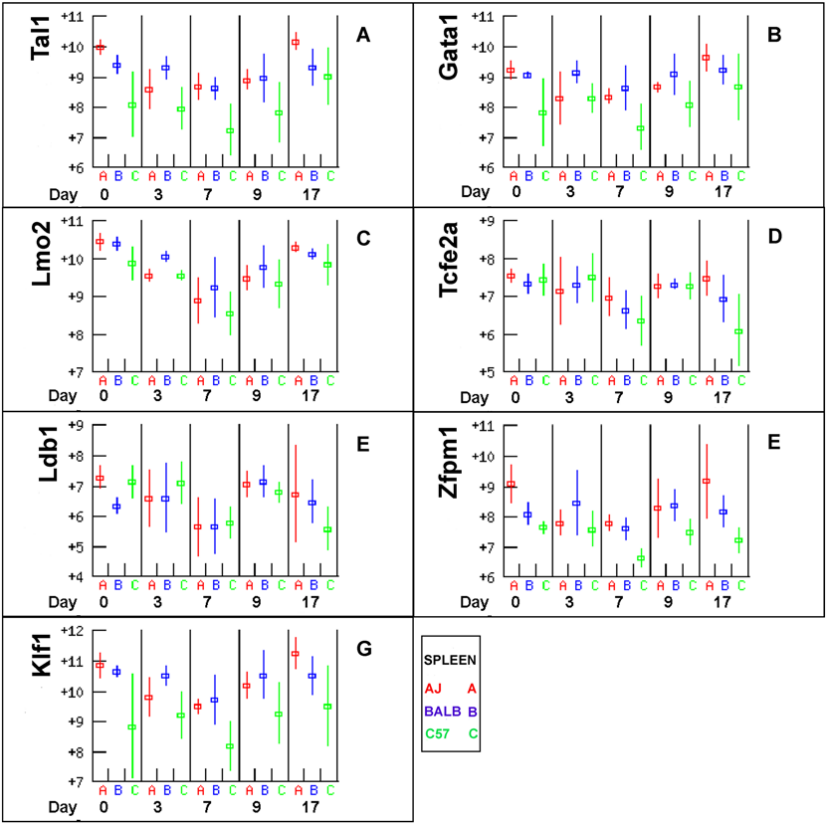
Transcription factors regulating erythropoiesis. *Tal1*, *Gata1*, *Lmo2*, *Ldb1*, *TcfE2a* and *Zfpm1* (*Fog1*) form a multimeric DNA binding complex, which regulates primitive haematopoiesis. All six genes were highly expressed and had similar patterns of expression consistent with co-ordinate regulation. *Klf1* is involved in erythroid cell proliferation and had similar levels and patterns of expression suggesting that it may be regulated by the same mechanisms. In all cases C57BL/6 mice tended to have the lowest levels of expression after day 3.

*Tal1*, *Gata1*, *Lmo2*, *Ldb1*, *TcfE2a* and *Zfpm1* (*Fog1*) form a multimeric DNA binding complex that regulates primitive haematopoiesis [41]. All six genes were highly expressed, declined in production in the spleen post infection and returned to near baseline levels by day 17, with the exception of *Ldb1*, *Zfpm1* and *Tcfe2a* in C57BL/6 (Fig 9). The transcription factor EKLF (*Klf1*) is involved in erythroid cell proliferation and has a similar expression profile suggesting that it might be co-regulated with the other six genes. C57BL/6 had lower levels of *Tal1*, *Gata1*, *Zfpm1* and *Kif1*, which are suggestive of lower levels of haematopoiesis in C57BL/6 particularly at later time-points. The similarity of their expression profiles is suggestive of co-ordinate regulation, which is consistent with the requirement for stoichiometric binding of the multimeric complex. The expression of *Gata2*, which acts earlier in erythropoiesis [41], did not change during infection and did not differ between strains (not shown).

### Erythrocyte structural proteins

**Figure 10:**
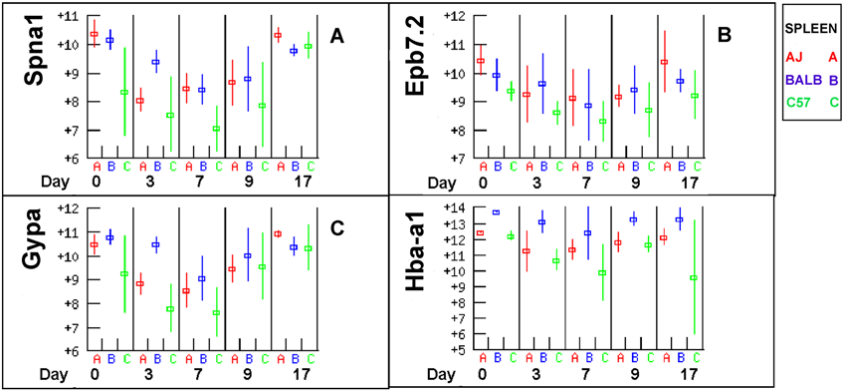
Erythrocyte structural proteins. Expression of erythrocyte structural protein genes followed the expression of their transcription factors (Fig 8) and C57BL/6 had lower expression levels than A/J or BALB/c.

Spectrin alpha and beta (*Spna1* and *Spnb1*), Glycophorin (*Gyp*a) and erythrocyte protein band 7 *Epb7.2* all declined in production in the spleen post infection but recovered by day 17 (Fig 10). In each case C57BL/6 had the lowest level of transcription consistent with relatively low levels of haematopoiesis. The expression of these genes tightly followed that of the regulatory genes described above (Fig 9) in both expression levels and in the decline from day zero to day seven followed by the recovery of expression to day 17. Haemoglobin-alpha (*Hba-a1)* was the most highly expressed gene in the spleen from day 0 and its expression changed little over the course of the infection but was 2–4 fold higher in BALB/c than A/J or C57BL/6 in both liver and spleen respectively (Fig 10). Haemoglobin beta expression was invariant and similar amongst all strains (not shown).

### Erythrocyte degradation

**Figure 11:**
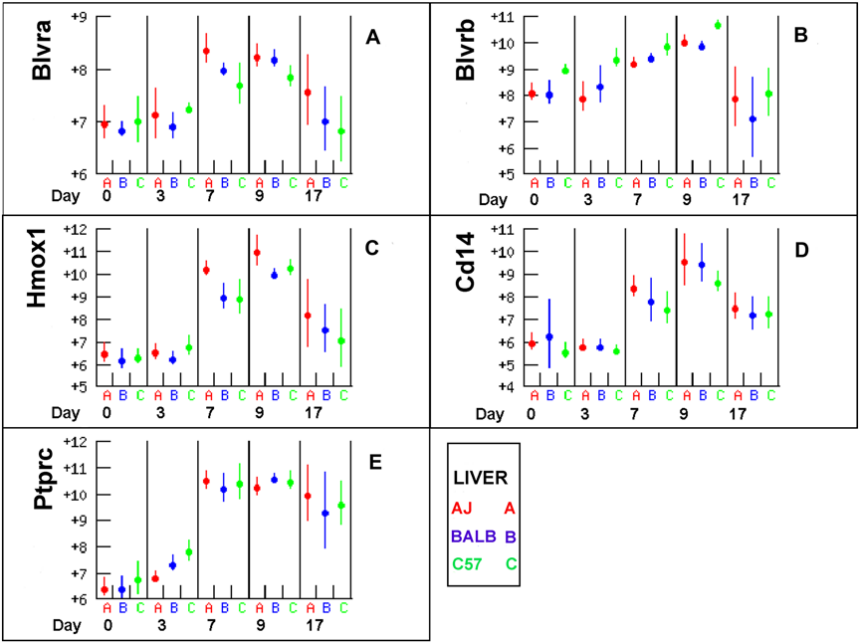
Erythrocyte degradation and leukocyte abundance. *Blvra* and *Hmox1*, which are involved in erythrocyte degradation, increased dramatically after infection but then declined to near baseline by day 17. *Cd14* and Cd45 (*Ptprc*) are markers of macrophage and leukocyte abundance respectively. Macrophages are the main cells involved in haemolysis and it appears that expression of *Blvra* and *Hmox1* was correlated with macrophage abundance. C57BL/6 did not have consistently higher expression of any of these genes, suggesting that higher or more chronic haemolysis is not the cause of the more chronic anaemia of this strain.

Biliveridin reductase a and b (BLVRA and BLVRB) and Heme oxygenase (HMOX1) are involved in degradation of erythrocytes and both *Blvra* and *Blvrb* expression increased 2–4 fold in the liver by day 9 post infection (Fig 11). HMOX1 cleaves the heme ring to form biliveridin which is reduced to bilirubin by BLVRA and BLVRB and which is then excreted in the bile. *Hmox1* expression increased 16 fold between days 3 and 9 in the liver from all strains, expression of all three genes declined by day 17. The expression of erythrocyte degradation genes correlated with expression of the pan-leukocyte antigens *Cd45* (*Ptprc*) and *Cd14*, which is primarily expressed on macrophages. Macrophages are the principal cells that destroy erythrocytes and trypanosomes are also cleared from the circulation by macrophages in the liver [42]. *Cd45* and *Cd14* expression in the liver was similar in all strains. Therefore although these data are consistent with the substantial increase in haemolysis after infection there is no evidence that different rates of haemolysis or haemophagocytosis are the cause of the more severe anaemia seen in C57BL/6. Furthermore expression of these genes declined to near baseline levels by day 17 in all strains suggesting that the more chronic anaemia of C57BL/6 is not a consequence of continuing haemolysis.

### Iron recycling by macrophages

Almost all iron for erythropoiesis is obtained by recycling existing stores. Given the evidence for the large increase in haemolysis and haem breakdown in the liver there should be evidence for a corresponding increase in iron recycling by liver macrophages. Hepcidin is known as the master regulator of iron storage in macrophages and its baseline levels are maintained by BMP/SMAD [43]. However during inflammation hepcidin appears to be primarily regulated by IL6 [44]. Hepcidin (HAMP) inhibits the release of recycled iron from macrophages by binding SLC40A1 (ferroportin) and targeting it for internalization and degradation [45], [46]. Expression of *Slc40a1* is also known to be repressed via a TLR4 mediated pathway after stimulation with LPS and IFNG, suggesting that *Slc40a1* expression is mediated by both hepcidin-dependent and independent pathways and that the latter may be more important in infections in which the TLR4 pathway is activated.

**Figure 12:**
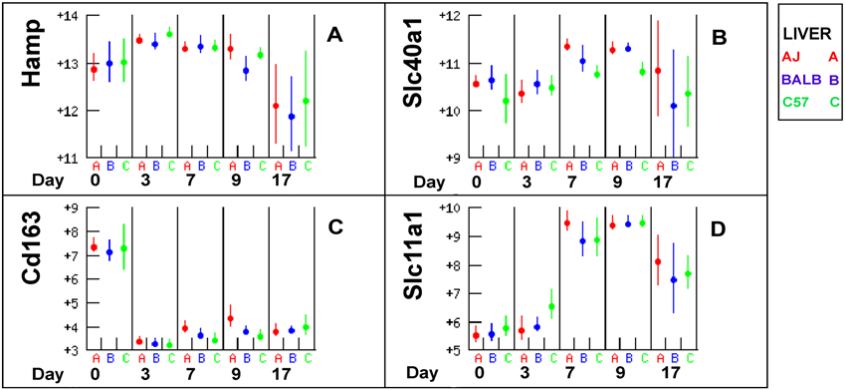
Expression of genes involved in iron recycling in the liver. *Hamp* is a negative regulator of *Slc40a1*, which exports iron from macrophages. Despite a decline in *Hamp* expression at day 17 there was no corresponding increase in *Slc40a1*. *Cd163* and *Slc11a1* are involved in uptake by macrophages of haem and molecular iron from the plasma. Both responded strongly to infection but the increase in *Slc11a1* may be for acquisition of iron for generation of oxidative stress for parasite killing rather than iron recycling.

*Il6* expression in the liver did not change (not shown). *Hamp* expression increased slightly post infection in all strains before declining to below baseline levels at day 17 (Fig. 12). *Slc40a1* expression in the liver followed that of *Hamp* (Fig. 12) and declined steadily in the spleen (Not shown). After export by SLC40A1, iron is loaded onto transferrin by Hephaestin, the expression of which remained constant until day 9 (Not shown). These data provide no persuasive evidence for substantial change in iron recycling after infection despite the evidence for a >10 fold increase in macrophage numbers. The relatively steady state expression of iron recycling genes compared with the large increase in expression of macrophage associated genes suggests that iron recycling by individual cells may have declined substantially.

### Iron Uptake by macrophages

CD163 expression is a scavenger receptor for haptoglobin and haem. *Cd163* expression was high at day zero and then declined >10 fold in both spleen and liver by day 3 to below the threshold of detection (Fig 12). In contrast expression of *Slc11a1* (*Nramp1*), which is a transporter of divalent cations including Fe^++^, increased about 20 fold between days three and seven (Fig. 12) following the expression of the macrophage and leukocyte markers CD14 and CD45 (Fig. 11). SLC11A1 is a macrophage protein and a metal ion transporter. It has a role in macrophage defense against microbial invasion and the increase in expression may be correlated with macrophage activation to kill parasites by oxidative stress as well as scavenging surplus Fe^++^ to reduce its abundance in the plasma.

## Discussion

Development of anaemia and other infection parameters were monitored during *T. congolense* infections in three inbred mouse strains and associations were made between anaemia development and gene expression profiles.

The characteristics of the anaemia in the mouse model used here were very similar to the anaemia in trypanotolerant and susceptible cattle and suggest that the causes of the anaemia are similar in both species. First, the kinetics of the anaemia development is similar in both species. The graph of haemoglobin in the three mouse strains studied, indicates two phases of anaemia development. The initial phase is characterized by a rapid decline in haemoglobin titres in all three mouse strains up to around day 10 post-infection, the time of the first peak of parasitaemia. In the second phase, haemoglobin levels continued to decrease in C57BL/6 mice although at a slower rate, while they recovered in A/J mice and even faster in BALB/c mice, accompanied by a simultaneous increase of transferrin levels. These two phases were also described in trypanosome infections in cattle, with the second phase starting after control of the first wave of parasitaemia. Stabilization and recovery of anaemia occurred in trypanotolerant, but not in trypanosusceptible breeds [6]. Second, in both species there is no evidence for a role of T cells in anaemia development. The preliminary experiment with CsA, which suppresses T lymphocyte functions, suggested that murine anaemia is not T cell-mediated. A similar lack of response to T cell suppression has been observed in cattle where depletion of CD4 or CD8 T cells in both susceptible and trypanotolerant breeds did not influence the severity of anaemia after *T. congolense* infection [14], [15]. Third, in cattle there is a degree of correlation between severity of anaemia and death. This also seems to be the case in C57BL/6 mice. Mortality started when RBC levels dropped below 60% of normal values, suggesting that severe anemia might be a contributory cause of death in this strain. However there was no correlation between anaemia and survival in the other two mouse strains. This has been observed previously with different combinations of parasites and mice [19], indicating that death in these strains is due to other causes, possibly related to the high parasitaemia levels. Fourth, the capacities to control parasitaemia and to limit anaemia in trypanotolerant cattle are the result of two unrelated mechanisms [15] and data in this paper and previous ones [17], [19] suggest that this is also the case in the mouse model. C57BL/6 mice had the lowest parasitaemia, yet developed the most severe anaemia, while A/J had high parasitaemia, but had better anaemia control.

An interesting observation was that the ability of A/J and BALB/c mice to recover from anaemia during infection was correlated with spleen size. BALB/c had the largest spleens and the highest expression of *Hba-a1* and the most rapid recovery from anaemia, while A/J had intermediate sized spleen and intermediate anaemia. The size of the spleen may correlate with haematopoietic capacity and account for the particularly rapid recovery of BALB/c mice. Spleen and liver size were significantly larger in female than male mice in the three strains, and differed in absolute size by about 20%. A study of twelve different mouse lines infected with *T. brucei* found females survived significantly longer than males in the seven lines with longest survival and that the difference was not X-linked [47]. However, in a study of A/J and C57BL/6 mice infected with *T. congolense* IL1180 [17], differences between the sexes in survival were observed but the effect differed in direction between strains and was not statistically significant (Nakamura personal communication), so any effect of sex on survival is likely to be small.

Several gene expression patterns measured in the arrays suggest higher erythropoietic activity in BALB/c compared to C57BL/6. Expression of *Epo* receptor, *Kit* and *Kit ligand* were consistently lower in C57BL/6. So was the expression of a number of erythroid structural proteins (spectrin and glycophorin), suggesting a lower density of erythroid precursors in tissues of C57BL/6. Further, expression of the transcription factors *Gata1*, *Lmo2*, *Fog1* (*Zfpm1*) and *Klf1* were lower in C57BL/6 than A/J and BALB/c, particularly at later time points, and the expression of *Ldb1*, *Zfpm1* and *Tcfe2a* had not returned to baseline levels by day 17, consistent with suppressed haematopoiesis in C57BL/6 mice in later stages. It is interesting to note that the more severe chronic anaemia of C57BL/6 mice also correlates with the lower number of Cobblestone Area Forming Cells that are in S phase in the femur of 7 day old C57BL/6 mice. These cells are a marker of the abundance of haematopoietic stem cells and A/J and BALB/c mice have approximately twice the numbers of them as C57BL/6 [48]. Consequently the more chronic anaemia of C57BL/6 mice may be a consequence of reduced numbers of haematopoietic stem cells and a more limited capacity to replace erythrocytes destroyed by haemolysis. In cattle, the capacity to recover from anaemia during an infection in genetically tolerant cattle, depends on the genetic background of the haematopoietic tissue, but not on that of lymphoid tissue, suggesting a role of erythropoietic responses in trypanotolerance [3].

The first phase of the anaemia development was characterized by an immediate and rapid decline in erythrocyte numbers in all three mouse strains (and in cattle breeds [3], [4], [5], [6]). Studies in infected cattle hinted that this may be related to phagocytosis of erythrocytes associated with the rising parasitaemia [7]. The 2–3 fold increase in Biliveridin expression and 16 fold increase in haem oxygenase expression that was observed between days 7–17 post-infection in the three mouse strains are consistent with substantially increased recycling of erythrocyte components in this period. Furthermore, A/J mice are known to be deficient in the C5 (Hc^0^) component of the complement cascade (http://jaxmice.jax.org/strain/000646.html). This is the component that forms the multimeric membrane attack complex that can destroy erythrocytes directly or more commonly by inducing phagocytosis of erythrocytes by macrophages. This would be expected to make A/J mice more resistant to the development of anaemia but since all strains developed anaemia at a similar rate in our experiment, the complement cascade may not be an important participant in this process.

The second phase of anaemia development differed markedly between the three mouse strains (and in previous studies between cattle breeds [3], [4], [5], [6], implying that this mechanism is dependent on genetic and host background. As discussed above, erythropoiesis and innate responses, but not acquired immune responses, may play an important role in this. Comparison of gene expression between the mouse strains could therefore give a clue as to which metabolic pathways might be responsible for the different phenotypes.

The lack of evidence for a response by *Ptp4a1* and *Tnfrs11a* to infection is consistent with previous reports of a blunted response to erythropoietin in the anaemia of chronic disease [49]. *Kif3a* and *Eif3a* have been shown to respond to EPO by increased expression, however *Kif3a* participates in multiple signaling pathways including *Wnt* and Sonic Hedghog [50]. *Eif3a* is required for maximal protein synthesis and is not a specific EPO response gene. Therefore although these genes have been found to respond to EPO [32], [51], the increase in expression may be due to the inflammatory stimulus rather than any EPO specific function.

The transcription factors *Tal1*, *Gata1*, *Lmo2* and *Fog1* (*Zfpm1*) all had lower expression in C57BL/6 than BALB/c consistent with more severe anaemia in C57BL/6. However, these transcription factors appeared to recover to preinfection levels by day 17 and correlated better with *Ifng* expression than with haemoglobin titre, so they may be responding to the acute phase inflammatory response rather than haemoglobin.

*Il6* has been proposed as a key link between inflammation and anaemia via its activation of hepcidin (*Hamp*), which acts as a negative regulator of intestinal iron absorption and macrophage iron release [51], [52]. Both *Il6* and hepcidin expression increased transiently and correlated with the transient anaemia observed in A/J and BALB/c mice, however by day 17 post infection hepcidin expression had declined to below pre-infection levels in all strains, indicating that hepcidin was unlikely to be responsible for the persistent anaemia in C57BL/6. Ferritin heavy chain *Fth1* expression declined 2–3 fold at day 17 post infection and ferritin abundance in the plasma returned to baseline by day 35 suggesting that there may not be a substantial increase in iron storage in ferritin in the chronic phase, however it is possible that iron is sequestered in haemosiderin deposits. In cattle *Il6* expression in blood mononuclear cells increased in all cattle breeds, but earlier in the susceptible than the tolerant breeds, suggesting its expression was correlated with disease severity [53].

The dramatic reduction in *Cd163* expression occurred before any other inflammatory markers, except the serum amyloid genes, had responded to infection. *Cd163* is exclusively expressed on monocytes and macrophages and is the scavenger for haem bound to haptoglobin. The decline in expression is particularly striking given the evidence for the substantial increase in macrophage numbers. Plasma haptoglobin levels have been found to rise rapidly in mice infected with *T. congolense* and this increase was the most sensitive marker of infection [54], the very early decline in CD163 expression could cause a reduction in haptoglobin uptake and the observed increase in plasma concentration. LPS and inflammatory cytokines such as IFN-γ are known to repress *Cd163* expression whereas anti-inflammatory cytokines such as IL4 and IL10 induce expression [55]. In the current study *Cd163* expression appeared to be an exquisitely sensitive marker of infection since expression declined >10 fold within three days of infection when parasites are hard to detect by microscopy and before any increase in expression of inflammatory cytokines. The GPI-anchor of the surface coat glycoprotein of *Trypanosoma cruzi* [51] and *Trypanosoma brucei* [56], [57] have been reported to have potent LPS-like properties, and the surface coat of *T. congolense* may have similar properties, so *Cd163* expression may be responding directly to parasite antigens. The down regulation of CD163 would be expected to lead to an increase of haem-haptoglobin complexes in the plasma, which has been observed [54], and a concomitant increase in oxidative stress which would have inflammatory and anti-parasitic effects [58]. In humans the haptoglobin-related protein HPR has been implicated in the sterile immunity of humans to *T. brucei brucei* by acting as a carrier for APOL1 [59].

The 16-fold increase in *Slc11a1* expression would be expected to be associated with a large increase in uptake of molecular iron. However *Slc11a1* expression after infection may be correlated with macrophage numbers and the control of oxidative stress rather than iron storage. The increase in abundance of transferrin in the plasma (Fig 5) in all strains but particularly A/J suggests that iron recycling is not impaired by the infection although the extensive haemolysis caused by the infection makes quantative studies difficult.

Overall there appears to be increased uptake of erythrocytes for degradation, reduced uptake of haptoglobin and decreased export of iron from the liver. The massive phagocytosis of erythrocytes is likely to exceed the available capacity of ferritin for storage and in such circumstances iron is removed from circulation by deposition as insoluble haemosiderin. The evidence for a large increase in erythrocyte degradation but no concomitant increase in iron export or ferritin production suggests that there may be a substantial increase in iron in haemosiderin, which is insoluble, and might restrict the availability of iron for erythropoiesis. An analysis of anaemia in C57BL/6 mice infected with *T. brucei* concluded that increased iron storage might be a cause of the chronic anaemia in that model [16]. However in the present study the expression of *Hamp* declined in all strains by day 17, which would be expected to permit an increase in iron recycling. Further studies are required to determine whether iron stored as insoluble haemosiderin is restricting availability for erythropoiesis as previously proposed [16] or if massive haemolysis and phagocytosis combined with reduced erythropoiesis is causing iron to be stored until required.

The strong association between chronic anaemia and inflammation would suggest that C57BL/6 mice might be maintaining a more persistent inflammatory state. However the relative resistance of C57BL/6 mice to *T. congolense* infection appears to be associated with the ability to switch from an initial Type 1 cytokine response (IFN-γ, TNF) to Type-2 cytokine production (first IL10, followed by IL4 and IL13). In contrast a continuing Type-1 cytokine response or an early mixed Type-1/Type-2/regulatory cytokine production confers susceptibility to trypanosome infections [60]. Consequently although it is quite likely that inflammation plays a role in the anaemia of C57BL/6 mice it seems unlikely that differences in inflammatory state, as measured by cytokine activity, can adequately account for the differences in anaemia after *T. congolense* infection.

### Conclusions

The data presented here showed that although A/J, BALB/c and C57BL/6 all developed anaemia in response to infection with *Trypanosoma congolense*, A/J and BALB/c mice were able to control the anaemia whilst C57BL/6 were not. Multiple genes involved in erythropoiesis responded to infection and correlated with the expression of genes that are markers of inflammation. The expression levels of almost all these genes returned to near baseline levels by day 17 post infection. This was consistent with the anaemia of A/J and BALB/c being regulated by the inflammatory process. The innate immune response may have been the major contributor to the inflammation associated with anaemia in these mouse strains since suppression of T cells with CsA had no observable effect. However the anaemia of C57BL/6 persisted long after the initial inflammatory stimulus possibly as a consequence of a more persistent inflammatory state in these mice. The transcription factors *Tal1*, *Gata1*, *Zfpm1* and *Klf1* all tended to be expressed at consistently lower levels in C57BL/6. The lowered expression of these genes may provide a valuable molecular marker for chronic anaemia and may be correlated with the small increase in spleen size and hence haematopoietic potential in C57BL/6 relative to A/J and BALB/c and may best account for the differences in anaemia in these mice.
